# Efficacy and Safety of Hypofractionated Radiotherapy With a Simultaneous Integrated Boost and With a Sequential Boost After Breast‐Conserving Surgery

**DOI:** 10.1002/cam4.70630

**Published:** 2025-03-23

**Authors:** Na Li, Yang Zhou, Jianting Wang, Yuwei Wang, Ruiyu Shao, Haifang Yang, Wei Xiong, Xuan Zheng, Xiaohong Wang

**Affiliations:** ^1^ Tangshan People's Hospital Tangshan Hebei China; ^2^ Department of Radiochemotherapy Tangshan People's Hospital Tangshan Hebei China

**Keywords:** breast‐conserving surgery, hypofractionated, radiotherapy, sequential, simultaneous

## Abstract

**Purpose:**

The objective of this study was retrospectively to compare the efficacy and safety of hypofractionated radiotherapy (HFRT) with a simultaneous integrated boost (SIB) or with a sequential boost (SB) after breast‐conserving surgery in patients diagnosed with early breast cancer.

**Methods:**

This study enrolled a total of 343 patients diagnosed with T1‐2N0‐1 breast cancer who had undergone breast‐conserving surgery followed by whole‐breast irradiation (WBI) without nodal irradiation, between March 2018 and April 2021. Out of 343 patients, 176 (51.3%) received HFRT‐SIB treatment, totaling 15 sessions, while the remaining 167 (48.7%) received HFRT‐SB treatment, totaling 18 sessions. Demographic characteristics, skin toxicity, radiation pneumonia, and myelosuppression, were compared in the two groups. Three‐year local progression free survival (LPFS) rates were determined using the Kaplan–Meier method and compared using the log‐rank test.

**Results:**

The median follow‐up time was 39.7 months (range 24.3–61.3 months). Toxicities rates did not differ significantly in the HFRT‐SIB and HFRT‐SB groups, including rates of grade 2 skin toxicity (14.8% vs. 13.8%, *p* = 0.721), Grade 2 radiation pneumonia (2.8% vs. 3.6%, *p* = 0.355), grades 1, 2 and 3 myelosuppression (12.5%, 5.7% and 1.1%, respectively, vs. 9.6%, 7.8%, and 1.2%, respectively; *p* = 0.744). Three‐year cumulative LPFS rates were similar in the HFRT‐SIB and HFRT‐SB groups (99.3% vs. 98.6%, *p* = 0.52). Regional nodal recurrences were observed in one patient in the HFRT‐SIB group (after 27.4 months) and in two patients in the HFRT‐SB group (after 29.4 and 56.4 months), and a local recurrence was observed in one patient in the latter group after 36.0 months. One patient in the HFRT‐SIB group was diagnosed with distant metastases to bone, and one patient in the HFRT‐SB group was diagnosed with distant metastases to the liver.

**Conclusion:**

Similar efficacy and safety of HFRT‐SIB and HFRT‐SB after breast‐conserving surgery in patients with early‐stage (T1‐2N0‐1) breast cancer. Longer‐term follow‐up is required to further compare their efficacy.

## 1 Background

Breast‐conserving surgery, followed by adjuvant radiotherapy is the standard treatment regimen for patients diagnosed with early breast cancer. Four randomized trials of early breast cancer patients in the United Kingdom and Canada between 1986 and 2002 have demonstrated that hypofractionated radiotherapy schedules are as safe and effective as conventional fractionation [1, 2, 3, 4]. Guidelines unanimously recommend hypofractionated radiotherapy for patients after early breast‐conserving surgery. But, no consensus has yet been reached for specific segmentation doses and modes for patients who require tumor bed supplementation.

To date, there are no randomized trials comparing hypofractionated radiotherapy with or without the simultaneous integrated boost (SIB) technique. A prospective non‐randomized trial found that local control rates were outstanding in patients who underwent conventionally fractionated whole‐breast radiotherapy with an SIB to the tumor bed [5]. Compared with hypofractionated whole body irradiation (HF‐WBI) plus sequential boost (SB) radiotherapy, the combination of HF‐WBI with SIB can further shorten the treatment time to about 5 days [6, 7, 8]. These results suggested that incorporating SIB into HF‐WBI is feasible, but that further studies were needed to optimize dosage to the tumor bed.

The placement of titanium clips on the tumor bed can simplify SIB. The present study retrospectively compared the efficacy and safety of HFRT‐SIB with HFRT‐SB in early breast cancer patients after breast conserving surgery. Safety outcomes specifically evaluated included rates of skin toxicity, radiation pneumonia, and myelosuppression.

## 2 Materials and Methods

### 2.1 Study Cohort

This retrospective study enrolled patients with early Stage I–II breast cancer who received radiotherapy after breast‐conserving surgery at our institution between March 2018 and April 2021. All patients met the inclusion criteria for the Z0011 study [9]. Patients were included if they were female; aged > 18 years; had a Karnofsky performance status (KPS) ≥ 80%; were diagnosed with pT1‐2, pN0‐1, M0 breast cancer; underwent breast cancer surgery plus axillary lymph node dissection (ALND) or sentinel lymph node biopsy (SLNB); and if postoperative pathology showed invasive carcinoma with negative resection margins. Patients were excluded N1 tumors, who requiring radiotherapy to the regional lymph node; if they had bilateral or multifocal breast cancer; breast cancer combined with other malignant tumors; ductal carcinoma in situ; if they received neoadjuvant chemotherapy or nodal irradiation; or if they had a history of chest radiotherapy. The Study has obtained written informed consent from the patients or their family, and is conducted with the approval of the medical ethics committee.

### 2.2 Radiotherapy

CT simulator positioning: The patient lies in a supine position on the breast support frame. Surface markings are made according to the positioning laser lines. The breast and scar areas are marked with lead wire. The patient breathes calmly, and a CT scan is performed, with the images transmitted to the treatment planning system. Tumor‐bed included seroma, surgical changes, and metal clips, and preoperative MRI also need to reference. The clinical target volume (CTV) included the entire soft tissue of the breast up to the deep fascia, whereas the planned target volume (PTV) included a CTV that is uniformly enlarged by 5 mm and PTV‐boost with a uniform margin of 15 mm around the tumor bed. Patients were evaluated by postoperative positioning CT and the surgical scar. The prescribed dose covering 95% of the PTV. The tumor‐bed boost was delivered to 100% isodose via enface electrons. The radiotherapy planning system uses Pinnacle 9.10. The accelerator equipment selected is Varian True Beam. The organs at risk (OAR), including the heart, lungs, contralateral breast, and spinal cord, are all within the normal irradiation range.

Both groups of patients received hypofractionated radiotherapy with the whole breast radiation dose of 43.5 Gy in 15 fractions. The tumor bed dose was 49.5 Gy in 15 fractions and for the simultaneous integrated boost group (HF‐SIB) and 8.7 Gy in 3 fractions and for sequential boost group (HF‐SB).

### 2.3 Other Systemic Treatments

Chemotherapy was administered to high‐risk patients according to the National Comprehensive Cancer Network guideline. Most patients received taxane‐based chemotherapy regimens, with or without anthracyclines. Hormonal therapy for patients with estrogen and/or progesterone receptor‐positive tumors, and anti‐human epidermal growth factor receptor 2 (HER2)‐targeted therapy recommended for patients with HER2‐positive tumors.

### 2.4 Follow‐Up

During follow‐up visits, the oncological and toxicological outcomes will be evaluated during RT; at 1, 2 weeks and 3, 6 months after radiotherapy; every 6 months for the first 5 years, and then annually. The follow‐up content includes history and physical exam, complete blood cell count, chest CT, regional nodal ultrasonography, ultrasonography/CT/MRI for live. All recurrences should be confirmed by histology or cytology if possible.

Local progression free survival (LPFS) was defined as the time from surgery to recurrence of ipsilateral breast and/or regional lymph nodes. Myelosuppression was categorized according to the National Cancer Institute's Common Terminology Criteria for Adverse Events version 5.0 (CTCAE 5.0), whereas acute skin toxicity and radiation pneumonitis were graded according to the Radiation Therapy Oncology Group (RTOG).

### 2.5 Statistical Analysis

The chi‐squared test was used to compare baseline characteristics between the HFRT‐SIB and HFRT‐SB cohorts, whereas continuous variables were compared by median tests. LPFS estimated using Kaplan–Meier method and compared by log rank test. Statistical analysis was performed using SPSS 25.0. Two‐sided *p* < 0.05 was considered to indicate statistically significant differences.

## 3 Results

The study cohort consisted of 343 women with Stage I–II breast cancer who underwent breast‐conserving surgery followed by entire breast irradiation between March 2018 and April 2021. Of these 343 women, 176 (51.3%) underwent HFRT‐SIB, and 167 (48.7%) underwent HFRT‐SB. The clinical characteristics and treatments of the two groups were well balanced (Table 1). The median age at diagnosis was 50 years (range 26–76 years). A total of 237 patients (69.1%) were treated with chemotherapy (Table 2). In total, 279 of 343 patients (81.3%) were hormone receptor dependent, all those receiving hormonal therapy. In addition, 56 patients (16.3%) were positive for HER2, with all receiving targeted therapy with trastuzumab, with or without pertuzumab.

**TABLE 1:** Tumor and treatment characteristics.

	HFRT‐SIB	HFRT‐SB	*p*
	*n* = 176	*n* = 167	
Age (year)			
> 50	91 (51.7%)	74 (44.3%)	0.195
≤ 50	85 (48.3%)	93 (55.7%)	
Tumor site			
Left	80 (45.5%)	78 (46.7%)	0.829
Right	96 (54.5%)	89 (53.3%)	
Histology			
Invasive ductal	157 (89.2%)	153 (91.6%)	0.470
Other	19 (10.8%)	14 (8.4%)	
pT stage			
1	142 (80.7%)	135 (80.8%)	1.000
2	34 (19.3%)	32 (19.2%)	
pN stage			
0	160 (90.9%)	148 (88.6%)	0.593
1	16 (9.1%)	19 (11.4%)	
Tumor grade			
I	13 (7.4%)	7 (4.2%)	0.543
II	112 (63.6%)	116 (69.5%)	
III	28 (15.9%)	24 (14.4%)	
Unknown	23 (13.1%)	20 (11.9%)	
Molecular subtype			
Luminal A	103 (58.5%)	94 (56.3%)	0.592
Luminal B	39 (22.2%)	43 (25.7%)	
HER 2 over expression	9 (5.1%)	12 (7.2%)	
Triple negative	25 (14.2%)	18 (10.8%)	
Her‐2			
Positive	26 (14.8%)	30 (18.0%)	0.466
Negative	150 (85.2%)	137 (82.0%)	
Chemotherapy			
Yes	125 (71.0%)	112 (67.1%)	0.483
No	51 (29.0%)	55 (32.9%)	

**TABLE 2:** The details of chemotherapy options.

Chemotherapy	HFRT‐SIB (*n* = 176)	HFRT‐SB (*n* = 167)	*p*
Chemotherapy regimen			
AC‐T	47 (37.6%)	53 (46.9%)	0.468
AC	13 (10.4%)	10 (8.8%)	
TC	50 (40.0%)	41 (36.3%)	
others	15 (12.0%)	9 (8.0%)	
Chemotherapy cycles			
4	53 (42.4%)	44 (38.9%)	0.353
6	24 (19.2%)	16 (14.2%)	
8	48 (38.4%)	53 (46.9%)	

### 3.1 Local Relapse and Distant Metastasis

Local and regional nodal recurrence was defined as ipsilateral breast tumor recurrence and/or ipsilateral medial breast, supraclavicular and axillary lymphatic recurrence. The median follow‐up in the entire patient cohort was 39.7 months, during which four patients developed local relapse. The median follow‐up in the HFRT‐SIB group was 34.9 months, during which time one patient (0.6%) experienced local recurrence. In comparison, the median follow‐up in the HFRT‐SB group was 43.0 months, during which three (1.8%) patients developed local recurrence. In addition, one patient in the HFRT‐SIB group was diagnosed with distant metastases to bone, and one patient in the HFRT‐SB group was diagnosed with distant metastases to the liver (Table 3).

**TABLE 3:** Local relapse and distant metastasis in the two groups.

	HFRT‐SIB (*n* = 176)	HFRT‐SB (*n* = 167)
Local recurrence	0	1
Regional nodal recurrence	1	2
Distant metastases	1	1

### 3.2 Survival Outcomes

**FIGURE 1:**
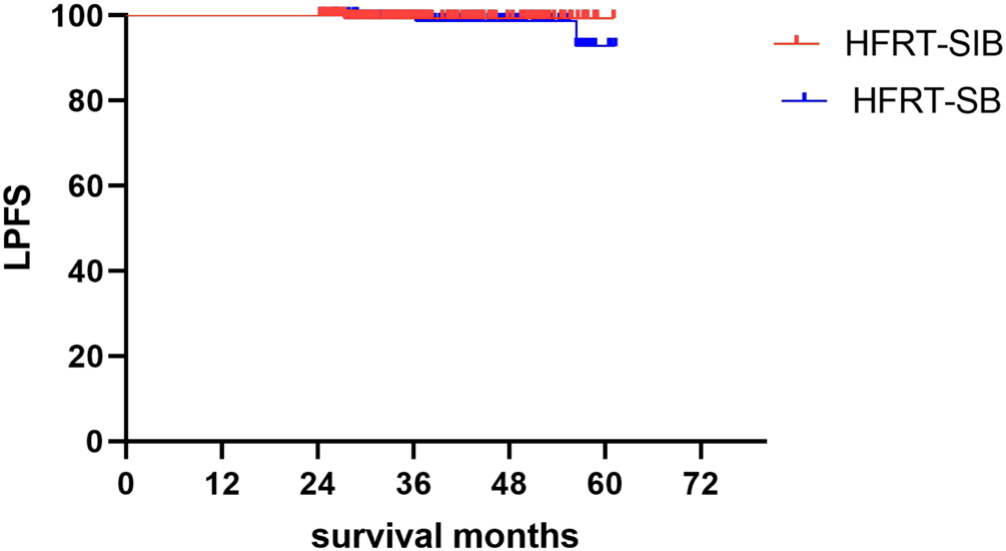
Kaplan–Meier analysis of 3‐year cumulative LPFS rates in the two groups.

Kaplan–Meier analysis showed that the 3‐year cumulative incidence of LPFS was 99.3% in the HFRT‐SIB group and 98.6% in the HFRT‐SB group (*p* = 0.52). (Figure 1).

### 3.3 Toxicity

None of the patients in either group experienced Grade 3–5 skin toxicity during or after radiotherapy. The incidence of Grades 0, 1 and 2 acute skin toxicity were 35.2%, 50.0%, and 14.8%, respectively, in the HFRT‐SIB group and 31.7%, 54.4%, and 13.8%, respectively, in the HFRT‐SB group, with no statistically significant between‐group differences.

The incidence of Grade 1 (asymptomatic, imaging findings only) and Grade 2 (symptomatic) radiation pneumonitis was 54.5% and 2.8%, respectively, in the HFRT‐SIB group, and 46.7% and 3.6%, respectively, in the HFRT‐SB group, with non‐significant differences between groups. None of the patients in either group experienced Grade 3 or higher pulmonary toxicity.

Maximum grades of immunosuppression were evaluated by routine blood tests before, during and after radiotherapy. The rates of Grades 1, 2, and 3 myelosuppression were 12.5%, 5.7%, and 1.1%, respectively, in the HFRT‐SIB group and 9.6%, 7.8%, and 1.2%, respectively, in the HFRT‐SB group, with no significant between‐group differences (Table 4). None of the patients in either group experienced Grade 4 myelosuppression.

**TABLE 4:** Maximum adverse reaction grades in the HFRT‐SIB and HFRT‐SIB groups.

Adverse	HFRT‐SIB (*n* = 176)	HFRT‐SB (*n* = 167)	*p*
Acute skin toxicity			
Grade 0	62 (35.2%)	53 (31.7%)	0.721
Grade 1	88 (50.0%)	91 (54.5%)	
Grade 2	26 (14.8%)	23 (13.8%)	
Pneumonitis			
Grade 0	75 (42.6%)	83 (49.7%)	0.355
Grade 1	96 (54.5%)	78 (46.7%)	
Grade 2	5 (2.8%)	6 (3.6%)	
Myelosuppression			
Grade 0	142 (80.7%)	136 (81.4%)	0.744
Grade 1	22 (12.5%)	16 (9.6%)	
Grade 2	10 (5.7%)	13 (7.8%)	
Grade 3	2 (1.1%)	2 (1.2%)	

*Note:* Two‐sided *p* < 0.05 was considered to indicate statistically significant differences.

## 4 Discussion

Whole‐breast radiotherapy following breast‐conserving surgery is the current standard of treatment for early stage breast cancer. Risk of recurrence must be evaluated in individual patients to determine the need for a booster dose to the tumor bed. Long term results have shown that 10‐year curative outcomes and local regional recurrence rates are similar in patients receiving hypofractionated and conventional fractionated radiation treatment, indicating that treatment with 13–16 fractions over 3–4 weeks was both safe and efficient [1, 2, 3, 10]. Another randomized controlled study showed that local recurrence and toxicity rates are similar in patients receiving conventional fractionated radiotherapy and hypofractionated radiotherapy with a tumor‐bed boost [11]. The optimal method of tumor bed supplementation to patients administered hypofractionated radiotherapy remains unclear. Tumor bed boost supplementation can be administered simultaneously and in an integrated manner with whole‐breast radiotherapy or sequential. Few studies to date have compared these treatment methods in patients with early breast cancer.

Simultaneous administration of an integrated boost in breast cancer patients has been reported to provide better dose homogeneity, to improve the biologically effective dose‐volume histogram and to reduce treatment time compared with SB [12, 13, 14]. A systematic literature review found that SIB plus hypofractionated radiotherapy was effective and safe, but required validation in prospective trials [6]. This research is important for understanding the impact of HF‐WBI with a simultaneous or subsequent HF‐boost on efficacy and safety in patients with early breast cancer. [Correction added on April 3, 2025 after first online publication. The sentence preceeding the last sentence has been deleted in this version.]

Inconsistent dosing of hypofractionated radiotherapy after breast conserving surgery may negative affect patient outcomes. One study evaluated the effects of administration of 40.5 Gy to the whole breast and 48 Gy to the tumor bed, both delivered in 15 fractions over 3 weeks [15]. Consistent with earlier findings [11], the HFRT‐SIB protocol in the present study consisted of administration of HFRT plus 3‐week hypofractionated SIB, with doses of 43.5 Gy to the whole breast and 49.5 Gy to the tumor bed, whereas the HFRT‐SB protocol consisted of WBI with 43.5 Gy in 15 fractions over 3‐weeks with a hypofractionated tumor‐bed boost of 8.7 Gy over 3 days.

Rates of Grades 0, 1, and 2 acute skin toxicity have been reported to be 12.1%, 83.3%, and 4.5%, respectively, in patients treated with intensity modulated radiation therapy with SIB group, and 10.0%, 71.7%, and 18.3%, respectively, in patients with conventional radiotherapy with SB group [16], with the rates of Grade 2 skin toxicity differing significantly in the two groups (*p* = 0.048). The present study, found that the rates of Grades 0, 1, and 2 skin toxicity were 35.2%, 50.0%, and 14.8%, respectively, in the HFRT‐SIB group and 31.7%, 54.5%, and 13.8%, respectively, in the HFRT‐SB group, with no patient having grade 3 or higher skin toxicity. Both the conventional fractionated radiotherapy in the above study and the hypofractionated radiotherapy in this study did not show any radiation skin toxicity of level 2 or higher. The difference is that the way the tumor bed is dosed during conventional fractionated radiotherapy, and the probability of developing Grade 2 radiation dermatitis is also different. The results of this study showed that there was no difference in the probability of radiation dermatitis between simultaneous and sequential dose addition methods in the context of hypofractionated radiotherapy. The probability of Grade 2 radiation dermatitis in this study was higher than that in the above study, and the reason for this may be the lack of post‐radiotherapy nursing education for patients in the primary hospitals, which we have found to be a problem, and we have actively taken measures to reduce the number of patients with Grade 2 radiation dermatitis in the past 2 years.

Although most studies of the side effects of hypofractionated radiotherapy for breast cancer have assessed skin toxicities, few evaluated rates of radiation pneumonia. One study reported that the rates of Grades 1 and 2 pneumonitis were 10.4% and 2.2%, respectively [11]. In comparison, the present study found that the rates Grades 1 and 2 pneumonitis were 54.5% and 2.8%, respectively, in the HFRT‐SIB group and 46.7% and 3.6%, respectively, in the HFRT‐SB group. As patient compliance increases, patients will undergo CT scans 3 months after the end of radiotherapy. The majority of patients are asymptomatic, with only minor imaging changes detected on review of a chest CT. This may be one of the reasons for the increased probability of Grade 1 radiation pneumonia in this study. Besides, the higher rate of Grade 1 pneumonitis observed in the present than in the previous study may have been related to radiotherapy planning, suggesting the need to optimize future radiotherapy protocols to reduce the rate of pneumonitis.

One study reported that the rates of Grades 1, 2, and 3 myelosuppression were 16.7%, 12.3%, and 3.5%, respectively, in patients receiving HFRT‐SIB and 30.0%, 21.1%, and 12.3%, respectively, in patients receiving conventional HF treatment. (*p* = 0.000) [17]. The present study yielded similar findings, with rates of Grades 1, 2, and 3 myelosuppression being 12.5%, 5.7%, and 1.1%, respectively, in the HFRT‐SIB group and 9.6%, 7.8%, and 1. 2%, respectively, in the HF‐SB group. None of these rates differed significantly, and no patient in either group experienced Grade 3 or higher myelosuppression. Patients with Grade 2 or higher myelosuppression are treated in‐hospital with drugs that increase leukocyte counts, and are discharged from the hospital when myelosuppression was controlled at Grade 0–1. Overall myelosuppression due to hypofractionated radiotherapy for breast cancer is safe and manageable.

An analysis of 348 patients who underwent breast‐conserving surgery and were followed‐up for a median 66.9 months showed that the 3‐year overall survival, disease‐free survival, and local recurrence‐free survival rates were 99.6%, 96.3%, and 97.7%, respectively [18]. In comparison, the present study found that the 3‐year LPFS rates were 99.3% in the HFRT‐SIB group and 98.6% in the HFRT‐SB group (*p* = 0.52). These findings indicate that HFRT after breast‐conserving surgery is both safe and effective, regardless of tumor bed dosage mode. Due to the short observation period, the results of the study may have some bias, which is a limitation of this study.

Hypofractionated radiotherapy after breast‐conserving surgery for breast cancer written into guidelines, but further exploration is needed regarding the dosing modality and the optimal fractionated dose for hypofractionated radiotherapy tumor beds after breast‐conserving surgery for breast cancer. This study retrospectively observed the efficacy and safety of hypofractionated radiotherapy after breast‐conserving surgery in an attempt to provide some clinical reference value. However, there are some limitations in this study. The first limitation that this is a single center retrospective study that lacks certain persuasiveness. Second, because the survival time of early breast cancer patients is long, the follow‐up time of this study is relatively short, and the research results may have some bias.

Nevertheless, this study showed that HFRT‐SIB and HFRT‐SB after breast‐conserving surgery were equally safe and feasible, with good toxicity profiles, in patients with early stage breast cancer. Conducting multicenter research may yield more convincing results. Studies that include longer follow‐up times are needed to further assess chronic complications and long‐term treatment outcomes.

## Author Contributions

**Na Li:** writing – original draft (equal). **Yang Zhou:** investigation (equal). **Jianting Wang:** investigation (equal). **Yuwei Wang:** investigation (equal). **Ruiyu Shao:** formal analysis (equal). **Haifang Yang:** data curation (equal). **Wei Xiong:** supervision (equal). **Xuan Zheng:** validation (equal). **Xiaohong Wang:** data curation (equal), project administration (equal), supervision (equal), writing – review and editing (equal).

## Ethics Statement

This study was carried, according to the revised Declaration of Helsinki (2013) and approved by the Institutional Review Board of Cancer Institute and Tangshan People's Hospital (Approval Number: RMYY‐LLKS‐2020‐027).

## Conflicts of Interest

The authors declare no conflicts of interest.

## Data Availability

The authors have nothing to report.
