# Correction to Efficacy and Safety of Hypofractionated Radiotherapy With a Simultaneous Integrated Boost and With a Sequential Boost After Breast‐Conserving Surgery

**DOI:** 10.1002/cam4.70882

**Published:** 2025-05-05

Li, N., Zhou, Y., Wang, J., Wang, Y., Shao, R., Yang, H., Xiong, W., Zheng, X. and Wang, X. (2025), Efficacy and Safety of Hypofractionated Radiotherapy With a Simultaneous Integrated Boost and With a Sequential Boost After Breast‐Conserving Surgery. *Cancer Med*, 14: e70630. https://doi.org/10.1002/cam4.70630

The penultimate sentence “The effect of the boost on the tumor bed is unclear, especially in patients receiving hypofractionated whole‐ breast radiotherapy. (delete…)” in the second paragraph of the discussion section needs to be deleted.

We apologize for this error.

